# An Important Point in the Examination of Sputum

**Published:** 1895-10

**Authors:** William G. Bissell

**Affiliations:** Bacteriologist, Department of Health, Buffalo, N. Y.


### AN IMPORTANT POINT IN THE EXAMINATION OF SPUTUM.

By WILLIAM G. BISSELL, M. D.
Bacteriologist, Department of Health, Buffalo, N. Y.

Much has been written as to different modes of procedure in the microscopical examination of sputum for the bacillus tuberculosis, such referring mostly to certain particular methods of staining and not touching on the point to which I wish to draw attention. The ordinary method, perhaps because of its being the easiest and not requiring special apparatus for its completion, of selecting particles to be mounted in tubercular examination is to select the lenticular cheesy masses contained in the sample, if such be present.

Microscopical examination of stained slide mounts of these masses, if the specimen be tubercular, will usually reveal the organism, but frequently will not and therein the difficulty lies.

It is a fact that the absence of the tubercle bacillus in a certain sample of sputum does not prove conclusively that it was from a non-tubercular source, for frequently the tubercular organism is absent from the sputum of consumptives for several days at a time, but the failure to find the organism when it is present seems hardly excusable.

If the technique of the operator is what it should be, and we will assume that it is, and if the bacillus tuberculosis is contained in the sample and yet not shown by examination, the error must lie in the only remaining factor, that is, the method by which the portion to be mounted was selected.

The only reliable method of examining sputum for tubercular infection is to select the portion to be mounted in such a manner as to be sure if the organism is contained in the sample it will be revealed in the mount.

This can be accomplished in the following way: As soon as the sample of sputum is received (and physicians should caution their patients to furnish bronchial and not pharyngeal secretion in a well-stoppered bottle), add to the sample about an equal volume of a 3 per cent. solution of sodium hydrate, shake well the mixture, and place one side to allow sedimentation. After a few hours draw from bottom of the bottle by means of a pipette what sediment may be present and centrifugate this sediment for about three minutes. If the bacillus tuberculosis be contained in the mixture, on centrifugation it will go to the bottom and will invariably be present in the first mount from the bottom of the tube.

As to methods of staining there are several good ones, but the one in my experience which gives the most uniform results is that of Liehl-Gabbot for cover glass preparation.

